# A Review of Clinical Disease Scoring Systems for Cicatricial Diseases of the Conjunctiva

**DOI:** 10.3389/fmed.2021.664572

**Published:** 2021-08-10

**Authors:** Hon Shing Ong, John K. Dart, Jodhbir S. Mehta

**Affiliations:** ^1^Corneal and External Diseases Department, Singapore National Eye Centre, Singapore, Singapore; ^2^Tissue Engineering and Cell Therapy Group, Singapore Eye Research Institute, Singapore, Singapore; ^3^Biomedical Research Centre at Moorfields Eye Hospital NHS Foundation Trust, National Institute of Health Research (NIHR), UCL Institute of Ophthalmology, London, United Kingdom; ^4^Duke-NUS Graduate Medical School, Singapore, Singapore; ^5^Corneal and External Diseases Department, Moorfields Eye Hospital NHS Foundation Trust, London, United Kingdom; ^6^School of Material Science and Engineering, Nanyang Technological University, Singapore, Singapore

**Keywords:** cicatrising conjunctivitis, cicatricial conjunctival diseases, conjunctiva, scarring, fornix depth, grading, mucous membrane pemphigoid, Stevens-Johnson Syndrome

## Abstract

Cicatricial conjunctival diseases (CCDs), are a diverse group of ocular surface diseases characterized by chronic scarring of the conjunctiva. These diseases can cause significant ocular morbidity. They are life-long once acquired and can be debilitating, painful diseases leading to visual loss. A recent international consensus of ocular surface disease experts have placed emphasis on the need of validated clinical disease scoring systems for CCDs, important for the objective evaluation of disease severity, outcomes of therapies, and longitudinal monitoring of disease. This review aims to describe the various published clinical disease scoring systems available for CCDs and evaluates the benefits and limitations of each system. It can be used as a guide for clinicians managing patients with CCDs and for researchers evaluating potential therapies in clinical trials.

## Introduction

Cicatricial conjunctival diseases (CCDs), are a diverse group of ocular surface diseases characterized by chronic scarring of the conjunctiva (1). As scarring is often the sequelae of chronic inflammation, these conjunctival conditions are thus also commonly known as cicatrising conjunctivitis (1). Severe CCDs can cause significant ocular morbidity. Patients with these diseases can suffer from chronic pain, which are often debilitating (1). Bilateral visual loss, caused by the complications of chronic ocular surface inflammation and scarring, has also been reported to affect as many as one in five patients with CCDs (1–4). As certain CCDs affect individuals of all ages, ranging from young children and healthy working adults to the older population, they can result in significant socio-economic burden (4).

Population based studies have indicated that ocular mucous membrane pemphigoid (OcMMP) is the major cause of CCDs in countries with predominantly Caucasian populations (4). The prevalence of OcMMP however, is relatively lower in Asian countries, where Stevens-Johnson Syndrome (SJS) / Toxic Epidermal Necrolysis (TEN) with ocular involvement is a common cause of CCD. In the developing world especially within the African continent, endemic *Chlamydia trachomatis* infections causing blinding cicatricial trachoma are common causes of CCD (5). It has been estimated that over 20 million people are actively affected by trachoma, with ~2 million suffering from severe visual impairment from the scarring complications of trachoma (5). There are over 30 other conditions reported to cause chronic conjunctival scarring (1). Other common causative conditions include traumatic injuries (e.g., chemical, thermal, and radiation), chronic allergic eye diseases, drug-induced conjunctival scarring, and other autoimmune diseases (e.g., Sjögrens syndrome, graft-versus-host disease) (1).

Scarring diseases of the conjunctiva pose significant diagnostic challenges (6). Although the pathogenic mechanisms through which different diseases result in CCDs vary significantly, most conditions result in similar consequent features at the ocular surface, which are often clinically indistinguishable (7). Examples of features of chronic conjunctival scarring include subepithelial fibrosis, forniceal foreshortening, symblepharon, ankyloblepharon, and loss of ocular motility. The effects of ocular surface damage caused by inflammation and scarring are also similar. These effects include dry eyes (punctate keratopathy), trichiasis or distichiasis, cicatricial entropion, lagophthalmos, recurrent corneal erosions or persistent epithelial defects, infectious keratitis, corneal opacification, corneal vascularisation, ocular surface keratinisation, corneal melts, and ocular surface failure. Although most ocular surface diseases can often be effectively managed through a systematic approach in identifying and treating these effects at the ocular surface (7), in CCDs, it can be important to identify the underlying disease (1). This is especially the case in progressive scarring diseases. An important example is OcMMP, which is a known progressive inflammatory and scarring disease (1, 8). Another example is a subset of patients with SJS / TEN, that develop autoantibody-positive or negative progressive conjunctival scarring similar to that in OcMMP, which may continue from the acute episode or develop years later (1). In these progressive CCDs, topical treatments are mostly inadequate and systemic immunosuppressive therapies are often required to control the disease process to avoid the development of sight-threatening complications (8). Due to diagnostic difficulties, delays in the commencement of treatment often occur, which can significantly affect the visual outcomes of these challenging diseases (4). Therefore, clinical assessment tools that are sufficiently sensitive to identify progressive CCDs and characterize the severity of disease are important to ensure the timely commencement of appropriate therapies.

Another challenge in the management of CCDs is the lack of effective therapies for these diseases. Current therapies for progressive CCDs are mostly reliant on empirical systemic immunomodulation. For example, depending on severity, the treatment of OcMMP include the use of corticosteroid therapies, immunosuppressive agents (e.g., dapsone, sulphapyridine, methotrexate, azathioprine, mycophenolate, and cyclophosphamide), biological therapies (e.g., rituximab), and intravenous immunoglobulins (8). However, adverse effects have been reported to occur in up to 30% of patients using these therapies (3, 8). Such adverse effects include life-threatening infections (e.g., activation of tuberulosis, viral hepatitis). Systemic corticosteroids are associated with loss of bone densities and Cushing's syndrome; various immunosuppressive agents can also result in blood dyscrasias, renal impairment, deranged liver function, and malignancies. Furthermore, treatment failures have been observed in up to 50% of patients, due to progression of scarring despite the control of inflammation (4, 8, 9). There is thus a need for more effective targeted therapies to treat CCDs.

Research in CCDs have been focused on the development of anti-inflammatory (10, 11) and more recently, anti-scarring therapies (12). For example, pre-clinical studies have reported the use of a repurposed drug disulfiram, an aldehyde dehydrogenase inhibitor, as a new potential therapy for the prevention and reversal of conjunctival scarring (12). To assess the therapeutic effects of such potential therapies for CCDs, a robust objective clinical assessment tool is required. Indeed, validated reproducible measurements of disease activity, conjunctival scarring, and the effects of ocular surface damage, are essential to evaluate the effects of new treatments in clinical trials (13).

This review aims to describe the various published clinical disease scoring systems available for cicatricial diseases of the conjunctiva and evaluates the benefits and limitations of each system. It can be used as a guide by clinicians managing patients with CCDs and by researchers evaluating potential therapies to treat such challenging conditions.

## Clinical Disease Scoring Systems for Cicatricial Diseases of the Conjunctiva

Various methods have been described to assess the severity of conjunctival scarring (Table 1).

**Table 1:** Summary of clinical disease scoring systems for cicatricial diseases of the conjunctiva.

	Mondino et al. (14)^*^	**Foster (15)^†^**	**Francis et al. (16)^‡^**	**Tauber et al. (17)^§^**	**Schwab et al. (18)**	**Rowsey et al. (19)**	**Sotozono et al. (20)*††***	**Kawakita et al. (21)**	**Williams et al. (22)**	**Reeves et al. (23)**	**Munyangango et al. (24)**	**Murrell et al. (25)^§§^**	Sharma et al. (26)^***^	**Ong et al. (27)**
Disease	OcMMP	OcMMP	OcMMP	OcMMP	Drug-induced cicatricial conjunctival disease and healthy controls	OcMMP	OcSJS	OcSJS and healthy controls	CCDs, and healthy controls	OcMMP	OcMMP	OcMMP	OcSJS	OcMMP and OcSJS
Study numbers	20 patients (40 eyes)	130 patients	17 patients (33 eyes)	75 patients (123 eyes)	Both eyes of 179 patients and 240 controls	4 patients (8 eyes)	73 patients (138 eyes)	5 patients and 20 controls	26 patients (51 eyes) with CCDs and 18 controls; 17 patients with identifiable causes: 10 OcMMP, 5 other dry eye diseases (3 Sjogren's syndrome), 2 OcSJS,	44 patients (79 eyes)	7 patients	-	200 patients (400 eyes)	109 OcMMP and 61 OcSJS
Validation	No	No	No	No	Yes	No	No	No	Yes	Yes	No	No	No	Yes
Parameters weighted	No	No	No	No	No	No	No	No	No	No	No	No	No	Yes
**Inflammation**														
Conjunctival hyperaemia			U				G				G	G	G	G
Limbitis														G
**Scarring**														
Subepithelial fibrosis		U	G	U							U	U		U^++^
Lower forniceal foreshortening	G	U	G	G	M^II^	M^**^		M^II^	M^II^	M^‡‡^	G	U		M^II^
Upper forniceal foreshortening			G					M^II^	M^II^			U		M^II^
Nasal forniceal foreshortening								M^II^						
Temporal forniceal foreshortening								M^II^						
Symblepharon		U	G	G			G				G	U	G	G
Ankyloblepharon		U		U							U	U		G^++^
Restriction in ocular motility			G											G^++^
**Effects of Inflammation and Scarring (morbidity)**														
Conjunctival keratinisation	U	U	G^#^	U									G	G^++^
Corneal keratinisation	U	U		U			G						G	G^++^
Visual acuity			G											
Schirmer's test			G											
Lagophthalmos			U											
Trichiasis / Distichiasis			G				G							G^++^
Entropion			G											
Corneal neovascularisation			G				G						G	G
Corneal opacification			G				G						G	G
Corneal infection			G											
Superficial punctate keratopathy			G^∧^				G							
Corneal epithelial defect							G						G	
Loss of Palisades of Vogt							G						G	
Corneal Conjunctivalisation							G						G	
Mucocutaneous junction involvement							G						G	
Meibomian gland involvement							G						G	
Punctal involvement							G						G	

*OcMMP, ocular mucous membrane pemphigoid; OcSJS, Stevens-Johnson Syndrome with ocular involvement; CCDs, cicatricial conjunctival diseases; G, graded; U, ungraded (present or absent); M, measured*.

* *Graded percentage forniceal foreshortening gives the four stages in Mondino system with keratinisation as components of stage 4*.

† *Presence or absence of subepithelial fibrosis, inferior forniceal foreshortening, symblepharon, ankyloblepharon (with keratinisation) gives the four stages in Foster system, respectively*.

‡ *Score based on clinical components with maximum score of 50, and a percentage derived by multiplying by two*.

# *Medial canthal keratinization*.

∧ *Fluorescein and Rose Bengal staining graded separately*.

§ *Staging system created using a combination of Mondino and Foster systems with addition of graded assessment of symblepharon*.

II *Using a fornix depth measurer or fornicometer*.

** *Conjunctival measurements using a ruler*.

++ *Score based on 13 components of three categories of ocular complications with maximum score of 39 for each eye*.

‡‡ *Slit beam horizontal and vertical conjunctival measurements*.

§§ *International panel of experts consensus publication*.

*** *Based on 12 components of three categories of ocular complications with maximum score of 53 for each eye*.

*Used in the study but left out of the final tool after analysis because of poor inter and intra-observer correlation*.

### Early Methodologies for the Assessment of Conjunctival Scarring

The initial clinical assessments of CCDs were introduced for patients with OcMMP. In 1981, Mondino and Brown introduced a method to grade the percentage shrinkage of the lower fornix (14). Reporting on 20 patients (40 eyes) with OcMMP, they described a four-stage grading assessment: stage 1: ≤ 25% shrinkage of conjunctival fornix; stage 2: 25–50% shrinkage of conjunctival fornix; stage 3: 75% shrinkage of conjunctival fornix; stage 4 (end-stage): obliterated conjunctival fornix and keratinization of conjunctival and corneal surfaces (14). In 1986's, Foster described a method of evaluating subepithelial fibrosis and the extent of symblepharon formation (15). Through a case series of 130 patients with OcMMP, they defined stage I as the presence of chronic conjunctivitis with subepithelial fibrosis; stage II was characterized by inferior fornix foreshortening (with features of stage I); stage III was defined by the appearance of a symblepharon (with features of stage II); and stage IV was defined as end stage disease with ankyloblepharon, severe sicca syndrome, and extreme ocular surface keratinisation (15). By combining these two methods, Tauber et al. subsequently introduced a system of grading CC (17); this system included assessing the loss of fornix depth, counting the number of symblephara, and estimating the percentage horizontal obliteration of the lower fornix by symblephara. The staging system proposed by Tauber et al. uses Foster's stages of I to IV as described above, with additional sub-divisions within stages II and III. In stage II, sub-divisions using letters a to d corresponded to the percentage shrinkage of the lower fornix similar to that described by Mondino and Brown: a: ≤ 25%; b: 25–50%; c: 50–75%; d: >75% loss of inferior fornix depth. In stage III, sub-divisions using letters a–d described the percentage of horizontal forniceal involvement by symblephara: a: ≤ 25%; b: 25–50%; c: 50–75%; d: >75% involvement by symblephara. The discrete numbers of countable symblephara are recorded in parentheses after the staging. For example, stage IIaIIIc(3) describes an eye with ≤ 25% inferior forniceal foreshortening with 25–50% horizontal forniceal involvement with three distinct symblephara.

The disadvantage of these methods of assessment is that they are based on clinical judgement of the extent of symblepharon and forniceal shrinkage. Thus, the assessments are largely qualitative and can be subjective. They are also limited to assessing scarring in the lower conjunctival fornix; upper conjunctival forniceal scarring, which can cause significant sight-threatening complications (e.g., upper lid entropion, lagophthalmos), are overlooked in these early methods. Moreover, in severe fibrotic disease, the extent of the fornix and thus the point of conjunctival reflection are often poorly defined. When the lower lid is everted, the forniceal conjunctiva also tends to become corrugated, and the tarsus may buckle. These factors make the assessment of forniceal shrinkage using the methods described by Modino and Brown, Foster, and Tauber technically difficult, as they require a view of the posterior lid surface. Despite their drawbacks, the Tauber and Foster methods have continued to be used by researchers in studies as they are simple enough to allow retrospective gradings from clinical records (6, 24).

### Objective Tools to Evaluate Conjunctival Fornices

**Figure 1:**
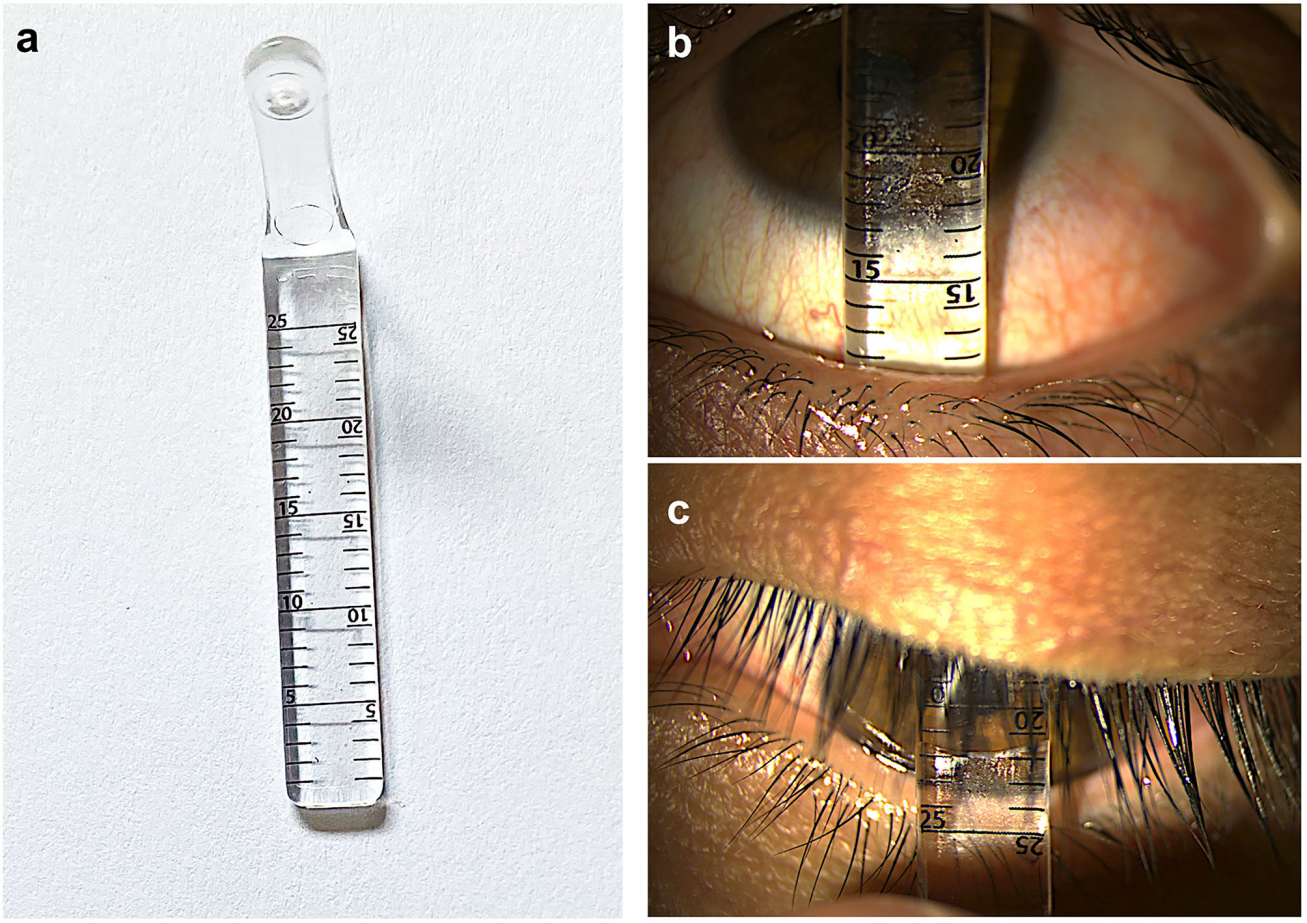
Measuring central conjunctival fornix depths. **(a)** An example of a fornix depth measurer (Scope Ophthalmics Ltd, United Kingdom). Note the millimetric markings in opposite directions to facilitate measurements in both upper and lower fornices; **(b)** to measure the central lower fornix depth, eyes are made to look in up gaze and readings taken at the eyelid margin; **(c)** to measure the central upper fornix depth, eyes are made to look in down gaze.

Recognizing the limitations of these early methods, various groups have subsequently introduced custom-made devices to facilitate the objective measurements of conjunctival fornices (18, 21, 22, 28, 29). Such devices include fornix depth measurers (FDMs), which allow the quantitative measurements of the lower, and in some devices, the upper fornix depths (Figure 1).

The first of such FDMs was described by Ivan Schwab et al. in 1992 (18). The authors described a customized metric ruler to objectively measure the inferior fornix depth (18). This ruler was used to monitor patients with drug induced CCDs (18). In this study, 179 glaucoma patients receiving topical glaucoma medications were measured to assess them for drug-induced CCDs; 420 control subjects with no history of ocular diseases were also measured (18). Using two-way analysis of variance (ANOVA), good inter-observer reliability and test-retest reliability were reported (0.901 and 0.945, respectively) (18). This was thus the first description of a validated objective tool to provide a quantitative measure of the inferior conjunctival fornix depth (18). Using measurements obtained from the control subjects, the authors were also able to provide age- and sex-stratified mean fornix depth values (18). Their data showed that there was a progressive shortening of mean fornix depths with advancing age; and that female participants tended to have shorter mean fornix depths, although this difference was less noticeable with advancing age (18). Such normalized data proved to be important in later research studies, allowing for sex- and age-adjustments whenever fornix depths were measured (22, 27).

Subsequently, two separate groups have developed other customized metric rulers to allow measurements of not only the lower, but also upper fornix depths which had previously been neglected as a result of poor access (21, 22). In 2009, Kawakita et al. designed a dull-edge steel rod (15 cm in length; 2 mm diameter) with a millimetric scale at each end (21). In this case series, the authors performed forniceal measurements in five Japanese patients with Stevens-Johnson syndrome and ocular involvement (OcSJS) and 20 healthy participants. This was the first study that provided “normal” mean fornix depth measurements for superior nasal and temporal fornices, inferior nasal and temporal fornices; and medial nasal and temporal fornices. The authors also estimated the overall area of conjunctival fornix to be ~909.6 mm^2^. However, due to the small sample size, stratification of normalized values based on age or sex could not be performed. This tool's inter-observer and test-retest reproducibility were also not reported.

In 2011, a separate group evaluated the upper and lower fornix depths of a heterogenous cohort of 26 patients (51 eyes) and 18 healthy control participants with various causes of CCDs (22). This group used a polymethymethacrylate FDM (22), similar to that described by Schwab et al. (18), but of increased length to allow upper fornix depth measurements. Within this series reported by Williams et al., 17 patients had an identifiable cause of cicatricial disease: 10 with OcMMP, two with OcSJS, and five with other severe dry eye diseases (including three with Sjogren's syndrome). Furthermore, the investigators implemented a correction factor for age by adapting age-specific lower fornix depths data published by Schwab et al. (18). They calculated the percentage loss of lower fornix using the equation: [(fornix depth (FD) age – FDM measurement)/FD age] × 100 = percentage (%) loss of fornix; the “FD age” values were derived from published age-specific lower fornix depths in normal eyes. Reproducibility of upper and lower fornix depth measurements were also established (22). Triplicate measurements of FDM readings of the central lower fornix depths by two examiners showed exact agreement in 86–89% of measurements; 100% of intra-observer measurements were within 1 mm for both examiners. The inter-observer agreement for lower fornix depth measurement was 86%, allowing for ± 1 mm variability. The intra-observer and inter-observer intraclass correlation coefficients (ICC) for lower fornix depth percentage foreshortening were 0.94 (95% CI 0.92–0.95) and 0.93 (95% CI 0.91–0.95), respectively. The investigators showed that upper fornix depth measurements were more variable. Triplicate measurements of FDM readings of the central upper fornix depths by two examiners showed exact agreement in 70–88% of measurements. Between the observers, there were agreements within 1 mm and 2 mm in 71 and 92% of the measurements, respectively. The intra-observer and inter-observer intraclass correlation coefficients for upper fornix depth percentage foreshortening were 0.92 (95% CI 0.89–0.94) and 0.89 (95% CI 0.81–0.93), respectively. This is the first study demonstrating the reproducibility of both upper and lower fornix depth measurements. Subsequent validation studies using a similar FDM was established in healthy eyes of Caucasian and South Asian subjects (28, 29).

In 2004, Rowsey et al. reported a new technique to quantify the degree of conjunctival scarring (19). The authors performed conjunctival stretching measurements in patients with OcMMP. Measurements (in mm) were taken from the lower limbus to the posterior edge of the retracted lower eyelid in three different positions of gaze: 5-o'clock position, 6-o'clock position, and 7-o'clock position. The sum of the three measurements represented the final value. By taking normal conjunctival measurements as 45 mm and the authors calculated percentage fornix foreshortening. They postulated that a shortening in 3 mm of fornix depth indicated disease progression. However, the investigators in this study did not account for the association of conjunctival anatomy to the age, sex, and ethnicity of patients. Furthermore, in this small case series of four patients, validation on reproducibility of measurements was not reported.

More recently, Reeves and associates described an alternative method aimed to quantify both lower vertical forniceal depth and horizontal diseased conjunctiva (23), similar to that described by Rowsey et al. (19). In this system, vertical fornix depth was measured using a slit-lamp, adjusting the slit-beam length to lie between the limbus at 6 o'clock and the start of the fibrosis with the lower lid gently retracted and the patient in upgaze. In their series, authors assumed a normal fornix depth of 10 mm. By subtracting 10 mm from the vertical fornix depth measurement and multiplying by 10, the percentage fornix foreshortening was calculated. For the horizontal conjunctiva affected by scarring, the total conjunctival width was first measured using a standard transparent ruler, along a horizontal line 2 mm above the start of the inferior scarred conjunctiva (if this is present), between the inner aspect of the nasal and lateral edges of the inferior posterior lid margin. The combined width of any symblephara was then subtracted from the total conjunctival horizontal width to give a percentage horizontal foreshortening. Reporting on 44 patients with OcMMP, they showed good levels of inter-observer agreement for both vertical and horizontal measurements (kappa statistic 0.86 and 0.80, respectively) (23); good correlation was also found between this system and the system described by Rowsey et al. (19). The authors concluded that both systems would give a complete grading of the severity of conjunctival scarring (23).

Nevertheless, limitations to systems described by Rowsey et al. and Reeves et al. exist. Firstly, the upper conjunctival fornix depth, an important measure of cicatrical disease as mentioned above, cannot be quantified using these methods. Secondly, lid laxity, which is common in eyes with OcMMP, can make these methods challenging by limiting sufficient stretch in the eyelid to achieve adequate measurements of the bulbar conjunctiva. Thirdly, unlike fornix depth measurements, data obtained from normal healthy eyes, stratified by age, sex, and ethnicity, is not available for these grading methods. Fourthly, the maximum length of the slit-beam on a standard slit-lamp is 8 mm. As the inferior fornix depths of healthy eyes are often > 10 mm, this limits the utility of the method described by Reeves et al. in quantifying fornix depths. Lastly, measuring only the forniceal depth or horizontal shortening from the bulbar conjunctival surface may not be ideal. Reeves et al. have remarked that the tarsal plate is relatively fixed anatomically and thus forniceal shortening on the posterior lid surface occurs in the conjunctiva below the inferior tarsus. This may not be entirely accurate. As described by Foster et al., subepithelial fibrosis over the tarsal plate is known to be an early clinical feature of CCDs (15). Such scarring changes along the tarsus often results in vertical contractures and shrinking of the tarsal length. This is well-described in CCDs (13). Using a FDM that uses the posterior lid margin as a point of reference allows the examiner to measure forniceal foreshortening below the tarsal plate, in addition to the shortening of the tarsus itself.

### Disease Activity, Damage, and Morbidity: More Comprehensive Ocular Surface Disease Scoring Systems (OSDISS)

**Figure 2:**
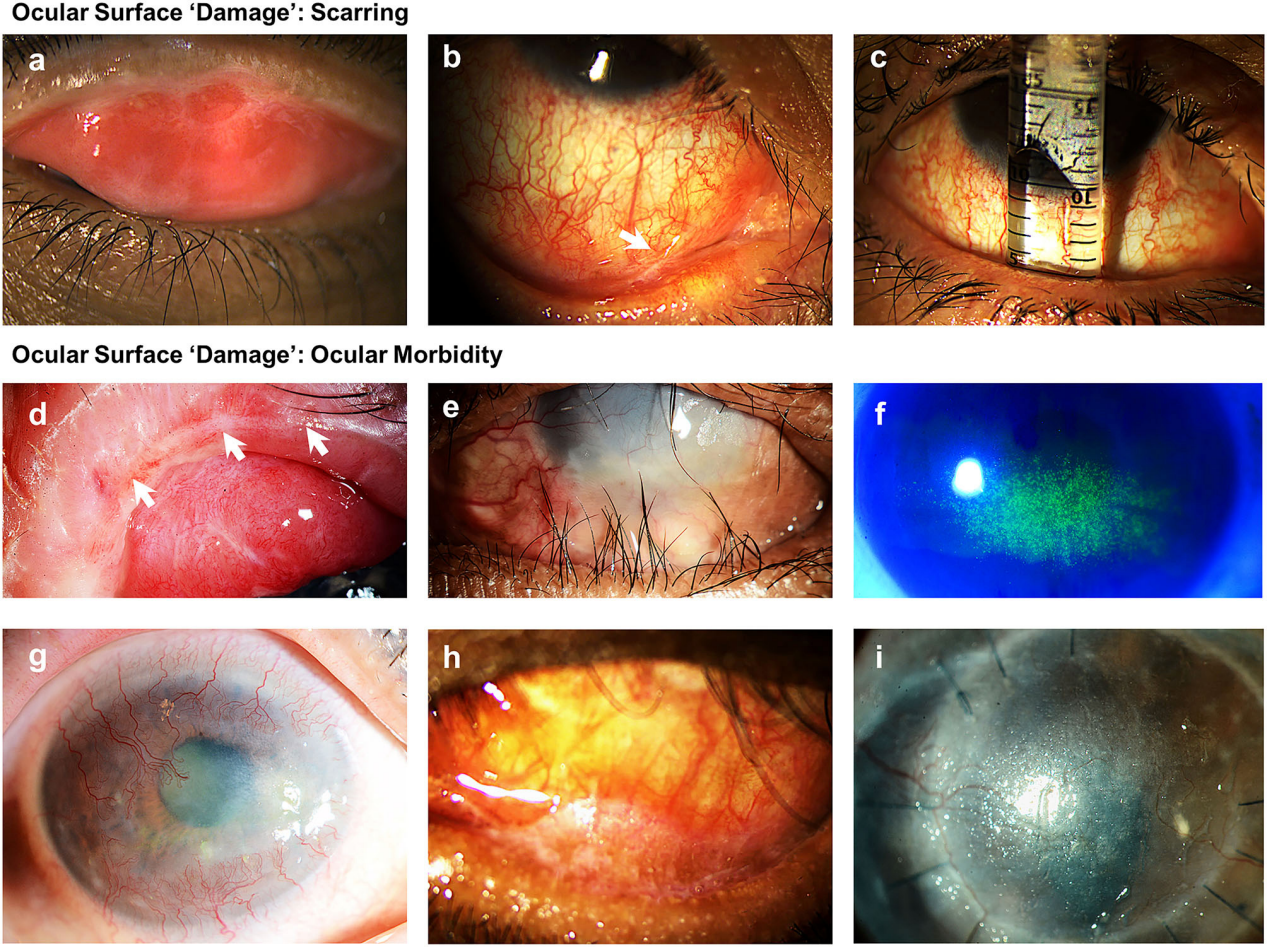
Examples of clinical manifestations of ocular surface “damage” related to scarring and ocular morbidity. **(a)** Subepithelial fibrosis; **(b)** presence of symblepharon; **(c)** conjunctival forniceal foreshortening illustrated using a fornix depth measurer; **(d)** disruption to meibomian glands and mucocutaneous junction; **(e)** trichiatic and distichiatic lashes; **(f)** superficial punctate keratopathy; **(g)** four quadrant central and peripheral corneal vascularization with opacification; **(h)** conjunctival and lid margin keratinization; **(i)** corneal keratinization.

In 2017, a steering group of international ocular surface diseases experts (OSDISS study group) published a set of core domains for the evaluation of ocular surface diseases (OSDs), through consensus using a modified Delphi technique (13). This document described the recognized clinical descriptors of OSDs and recommended that ocular surface manifestations should be classified into “*disease activity*” and “*damage*” (Table 2). “*Disease activity*” are clinical parameters that are the result of active inflammation, which can often be reversed with time or following treatments such as immunomodulation. Ocular surface “*damage*” represents clinical features that are irreversible, defined in the consensus document as persisting for over 6 months. These features result from changes in ocular surface anatomy, physiology, pathology, or function. In CCDs, examples of parameters describing “*damage*” are those related to conjunctival scarring, such as subepithelial fibrosis, forniceal foreshortening, symblepharon, and ankyloblepharon (Figures 2a–c) “*Damage*” also represents the longer-term sight-threatening effects or complications of ocular surface inflammation and scarring. These have also been described as “*ocular morbidity*” (27) (Figures 2d–i) The evaluation of ocular surface manifestations classed under “*ocular morbidities*” is important in determining the severity of disease, as the presence of these are often associated with poor visual prognosis (13). Some examples of clinical manifestations categorized under “*ocular morbidity*” include punctate epitheliopathy, corneal vascularisation, corneal conjunctivalisation, corneal opacification, and ocular surface keratinisation. In the consensus document, a need for a validated ocular surface disease scoring system (OSDISS) which evaluates both “*disease activity*” and ocular surface “*damage*” was emphasized (13). Such an OSDISS is important in clinical practice for the accurate detection of disease (diagnosis), evaluation of disease severity, prognostication of disease, and the objective monitoring of treatment response. It is also required for the standardization of research data collection, especially when evaluating potential therapies for CCDs.

**Table 2:** Categories of ocular surface manifestations in ocular surface disease scoring systems (OSDISS).

**Categories in ocular surface disease scoring systems (OSDISS)**	**Ocular surface manifestations**
Disease activity	• Conjunctival hyperaemia • Limbitis
Ocular surface damage: Scarring	• Subepithelial fibrosis • Forniceal shortening • Symblepharon • Ankyloblepharon • Restriction in ocular motility
Ocular surface damage: Morbidity	• Lagophthalmos • Trichiasis/Distichiasis • Entropion • Mucocutaneous junction involvement • Meibomian gland dysfunction • Punctal involvement • Tear deficiency • Punctate epitheliopathy • Corneal epithelial defect • Corneal neovascularisation • Corneal infection • Loss of palisades of Vogt • Corneal opacification • Corneal conjunctivalisation • Conjunctival keratinisation • Corneal keratinisation • Visual acuity

Studies described in this review have so far been focused on improving the detection and grading conjunctival scarring, caused by various CCDs. Grading systems introduced in these studies focus on one aspect of ocular surface “*damage*,” but have largely failed to account for “*disease activity*.” These grading systems tend to infer “*disease activity*” and progression by changes in the degree of conjunctival scarring. However, irreversible conjunctival scarring can often take weeks to years to develop, often as a result of uncontrolled inflammation. Thus, tools that can detect active disease and the severity of activity are crucial, to ensure timely control of inflammation and the prevention of irreversible ocular surface damage. Moreover, these systems also lack the important assessment of “*ocular morbidity*.”

The first documented OSDISS, that included the evaluation of parameters of both “*disease activity*” and ocular surface “*damage*,” was introduced by Francis et al. in 1990 (16). Through a case series of 17 patients with OcMMP, the authors used a defined grading schema which comprised 16 components of OSD manifestations. An ungraded absence or presence of conjunctival inflammation was included in the grading schema as an evaluation of “*disease activity*.” “*Damage*” in terms of conjunctival scarring was included as graded assessments of subepithelial fibrosis, upper and lower forniceal foreshortening, number of symblephara, and ocular motility. “*Damage*” in terms of *ocular morbidity* was evaluated as assessments in visual acuities (graded), fluorescein staining (graded), Rose Bengal staining (graded), Schirmer's test (graded), trichiasis (graded), entropion (graded), lagophthalmos (ungraded), corneal vascularisation (graded), corneal infection (graded), and medial canthal keratinisation (graded). This system gives a numerical grading score based on clinical components with a maximum score of 50, and a percentage derived by multiplying by two. Although comprehensive, this grading schema was never validated and thus has not been widely adopted by clinicians and researchers.

Munyangango et al. subsequently described a graded method for scoring “*disease activity*” in OcMMP. Each eye is divided into four quadrants and the degree of conjunctival hyperaemia graded on an eight point score (0.5, 1, 1.5, 2, 2.5, 3, 3.5, 4) (24). In this study, grading of conjunctival scarring was based on the system described by Tauber et al. (24). No other parameters of ocular surface “*damage*” or *ocular morbidity* were included (24). This system of conjunctival inflammation grading was later adopted within a systemic MMP scoring system published following consensus of an international panel of experts in bullous diseases (25). However, the recording of ocular surface “*damage*” in this system was ungraded (presence or absence) and poorly defined as “ocular scarring,” with no reference to specific clinical features of conjunctival scarring.

In 2007, Sotozono et al. published a grading system for patients with SJS / TEN (20). Unlike previously described assessment techniques, this system graded the severity of disease based on “*disease activity*” and the effects of cicatrisation (*damage*). It comprised 13 components within three categories of ocular complications. These included eyelid complications (meibomian gland involvement, trichiasis, mucocutaneous junction involvement, punctal involvement), conjunctival complications (hyperaemia and symblepharon formation), and corneal complications (punctate keratopathy, corneal epithelial defect, loss of palisades of Vogt, conjunctivalization, neovascularization, opacification, and keratinization). Each component was graded 0–3, depending on the severity of the complication. Through multivariable regression, the authors showed that their grading of corneal neovascularization, opacification, and keratinization had significant effects on logMAR visual acuities. Despite no data on inter- and intra-observer reproducibility, this system has been widely adopted in OcSJS research (30–32).

The system introduced by Sotozono et al., evaluating the chronic ocular complications of SJS, was more recently modified by Sharma et al. (26). To differentiate the more severe cases from the less severe ones, these authors expanded the grading of each ocular surface manifestation to give a maximum score of 5 (instead of 3). In this system, the authors showed that all 12 components of three categories used for grading, and total severity scores, correlated significantly with the CDVA of patients. Nonetheless, like the system proposed by Francis et al., the examiner using either Sotozono et al.'s or Sharma et al.'s method, needs to assess multiple clinical parameters, many of which are subjective.

To fulfill unmet needs as set out in the 2017 international OSDISS study group consensus (13), our group recently set out to design a concise, validated, semi-quantitative clinical severity assessment tool for CCDs (27). The aim was to create a much simpler grading system, that would still include components that measure inflammation or “*disease activity*” and ocular surface “*damage*” (scarring and ocular morbidity). Clinical manifestations of CCDs to be evaluated were chosen from previously described disease activity and damage indices for OcMMP and OcSJS (13, 16), and the observations of our cross-sectional and longitudinal OcMMP studies (6, 33). The original design of the assessment tool comprised of 12 components in three functional categories: (a) *inflammation grading* (bulbar conjunctival hyperaemia, limbitis), (b) *scarring grading* (subconjunctival fibrosis, limitation in ocular motility, upper and lower fornix symblephara, upper and lower central fornix depth measure), and (c) *ocular morbidity grading* (distichiasis, conjunctival and corneal keratinisation, corneal vascularisation, and corneal opacity). Through a rigorous validation exercise, the assessment tool was subsequently modified to include only components with good inter-observer and intra-observer (test-retest) agreements and components which showed low redundancy. The assessment of redundancy was performed by correlation analyses between each component and the other components, where poor to moderate correlations indicated that one component did not have the potential to adequately predict the presence or severity other components. Examples of components which showed low redundancy included upper and lower symblephara assessments, upper and lower fornix depth measurements, corneal vascularisation, and corneal opacity.

Of the 12 components, seven were found to have moderate to excellent levels of agreement: (a) *inflammation grading* (bulbar conjunctival hyperaemia), (b) *scarring grading* (upper and lower fornix symblephara, upper and lower central fornix depth measure), and (c) *ocular morbidity grading* (corneal vascularisation, corneal opacity). In our proposed clinical assessment tool, each of the seven components within each category have a graded scoring scale. A combined composite score (on a percentage scale out of 100) can then be calculated to provide the user with an overall assessment of disease severity.

Unlike published inflammation scoring schemes which have mostly used subjective assessments of conjunctival injection (20, 34), the method we introduced was guided by comparison to a standard panel of photographs and showed good inter-observer agreement (interclass correlation coefficient, ICC = 0.88, 95% CI 0.84 – 0.90). Good inter- and intra-observer agreements to quantify fornix foreshortening were also achieved with the use of a FDM, similar to that validated in previous studies (22, 28, 29). Furthermore, this is the first scoring tool that apportioned different weightage to certain OSD manifestations within the scoring system. This is considered important in determining disease severity. For example, within the morbidity category, opacities affecting the central cornea are given a proportionally higher weighted score, compared to opacities affecting the peripheral cornea.

Poor levels of agreements were observed in limitation in motility component (scarring category) and thus, this was left out of the final assessment tool. Due to their clinical importance, four components were left in the final tool, despite insufficient statistical data to show reproducibility. However, unlike the seven components that showed adequate levels of agreement, these four components are recorded but not scored and do not contribute to the overall composite disease severity score. These include ocular surface keratinisation (morbidity category), limbitis (inflammation category), distichiasis (scarring category), and subconjunctival fibrosis (scarring category). Although there was inadequate agreement in ocular surface keratinisation, it was retained as it is a known indicator of ocular surface disease severity and poor visual prognosis. Limbitis was absent in the study participants, and thus adequate levels of agreement could not be determined. However, limbitis was left in the final assessment tool as it is an important marker of severe ocular surface inflammation and a feature of poor visual prognosis. Similarly, subconjunctival fibrosis, an important early diagnostic sign of CCDs was found in 98% of participants and thus was left in the final tool. Inadequate levels of agreement could not be determined also for distichiasis; however, this was left in as an important marker of ocular surface fibrosis, especially in OcSJS.

The final tool proposed thus comprised of 11 components:

a) *Inflammation grading*
  i. Bulbar conjunctival hyperaemia (scored and contribute to composite score)
  ii. Limbitis (recorded but not scored)

b) *Scarring grading*
  i. Upper and lower fornix symblephara (scored and contribute to composite score)
  ii. Upper and lower central fornix depth measure (scored and contribute to composite score)
  iii. Subconjunctival fibrosis (recorded but not scored)

c) *Ocular morbidity grading*
  i. Corneal vascularisation (scored and contribute to composite score)
  ii. Corneal opacity (scored and contribute to composite score)
  iii. Distichiasis (recorded but not scored)
  iv. Ocular surface keratinisation (recorded but not scored)

In our final proposed assessment tool for CCD, each category comprises important measures required for clinical evaluation and for clinical trials. This tool can be used in its entirety, to provide an overall disease severity score. Alternatively, the different categories (inflammation, scarring, or morbidity grading) can be used independently.

The scoring system used in our clinical assessment tool has been compared to the system introduced by Sotozono et al., in a cohort of patients with OcSJS. Good correlation and agreement were found between the two grading systems [Pearson *r* 0.93, *p* < 0.001]. This is despite a significant reduction in the number of components graded, from 13 in the Sotozono et al. grading tool to 7 in our tool. Our validated clinical assessment tool for CCDs, which uses a minimal number of clinical components to evaluate the severity of disease has since been prepared in a form ready for use in clinical practice and clinical trials.

## Conclusion

This review summarizes the development of the scoring systems available for CCDs. It describes the deficiencies of some of the previous grading tools, which focused mostly on ocular surface scarring caused by CCDs. The importance of scoring systems that make distinctions between disease “*activity*” and “*damage*,” is increasingly recognized (13, 35). An overview of such scoring systems has thus been provided in this review, with the benefits and limitations of each system. We hope that this review will serve as a useful guide for ophthalmologists and researchers when choosing an assessment tool for clinical practice or clinical trials.

## Author Contributions

HO, JD, and JM: conceptualization, writing draft, review, and editing. HO: data curation. HO and JM: funding acquisition. All authors approved the manuscript.

## Conflict of Interest

The authors declare that the research was conducted in the absence of any commercial or financial relationships that could be construed as a potential conflict of interest.

## Publisher's Note

All claims expressed in this article are solely those of the authors and do not necessarily represent those of their affiliated organizations, or those of the publisher, the editors and the reviewers. Any product that may be evaluated in this article, or claim that may be made by its manufacturer, is not guaranteed or endorsed by the publisher.
